# MicroRNA Buffering and Altered Variance of Gene Expression in Response to *Salmonella* Infection

**DOI:** 10.1371/journal.pone.0094352

**Published:** 2014-04-09

**Authors:** Hua Bao, Arun Kommadath, Graham S. Plastow, Christopher K. Tuggle, Le Luo Guan, Paul Stothard

**Affiliations:** 1 Department of Agricultural, Food and Nutritional Science, University of Alberta, Edmonton, Alberta, Canada; 2 Department of Animal Science, Iowa State University, Ames, Iowa, United States of America; Niels Bohr Institute, Denmark

## Abstract

One potential role of miRNAs is to buffer variation in gene expression, although conflicting results have been reported. To investigate the buffering role of miRNAs in response to *Salmonella* infection in pigs, we sequenced miRNA and mRNA in whole blood from 15 pig samples before and after *Salmonella* challenge. By analyzing inter-individual variation in gene expression patterns, we found that for moderately and lowly expressed genes, putative miRNA targets showed significantly lower expression variance compared with non-miRNA-targets. Expression variance between highly expressed miRNA targets and non-miRNA-targets was not significantly different. Further, miRNA targets demonstrated significantly reduced variance after challenge whereas non-miRNA-targets did not. RNA binding proteins (RBPs) are significantly enriched among the miRNA targets with dramatically reduced variance of expression after *Salmonella* challenge. Moreover, we found evidence that targets of young (less-conserved) miRNAs showed lower expression variance compared with targets of old (evolutionarily conserved) miRNAs. These findings point to the importance of a buffering effect of miRNAs for relatively lowly expressed genes, and suggest that the reduced expression variation of RBPs may play an important role in response to *Salmonella* infection.

## Introduction

MicroRNAs (miRNAs) are post-transcriptional regulators [1]–[3]. The proposed functions of miRNAs can be broadly classified into two categories: tuning and buffering the expression of their target genes [4]–[7]. The role of miRNAs to fine-tune the expression levels (i.e., reset the mean expression levels) of their targets to promote cell differentiation at specific times during developmental stages is well known [4]. For example, miR-150 targets the *Myb* gene, which then regulates the formation of pre-B and B1 cells in the mouse immune system [8]. A high level of miR-150 holds *Myb* expression in check, preventing the pre-maturation of pro-B cells into pre-B cells.

The buffering effects of miRNA, however, are contentious, mostly deriving support from simulation studies and only a few cases with experimental validation. For example, a simulation study showed that miRNAs within an incoherent feed forward loop (IFFL) can reduce the noise during target gene expression [9]. Another simulation study suggested that noncoding RNAs can be more effective than transcriptional factors (TFs) in filtering input noises [10]. miRNA-mediated IFFLs are RNA-based, rather than protein-based, and hence the mechanism of action is faster and more cost-effective (requires less energy) [10]. A case study supporting the buffering role of miRNAs showed that miR-7 can stabilize the developmental process against temperature perturbation in Drosophila by buffering the expression of *yan* and *ato* [11]. Another study, also done in Drosophila, showed that expression of miR-8 is essential to regulate the atrophin gene within the required expression range [12]. However, previous genome-wide studies have reached mixed conclusions on buffering effects of miRNAs in expression variation within and between species. By comparing data from mammal and fly species, Cui *et al.* found that the cross-species expression variation of miRNA targets is significantly lower than that of other genes [13]. miRNA targets have also been shown to be significantly enriched in stably expressed genes in human [14]. However, Lu *et al.* [15] found increased variation of miRNA target gene expression compared with non-targets among the natural human population.

One explanation for the seemingly inconsistent results with respect to miRNA buffering is that it only operates under certain conditions. The buffering role of miRNAs may not be apparent under normal, and/or environmentally stable conditions but becomes evident under environmental perturbations [4]. Exposure of cells to pathogenic infections or to any environment that reduces cell viability or fitness can be considered perturbations. Multi-cellular organisms have the capacity for internal homeostasis, thus they can buffer extracellular changes to minimize intracellular alterations [16]. miRNAs may act to provide stability to some key molecular networks to maintain the internal homeostasis, which ensures the cell’s function, fitness and survival under environmental perturbations.

Furthermore, the degree to which buffering effects operate on genes with different expression levels is still poorly understood.

From an evolutionary perspective, it has been hypothesized that the dual functions of miRNAs may represent two stages in their evolution [4]. Compared to conserved miRNAs, young (less conserved) miRNAs are not likely to reset the expression level of several targets, but tend to increase fitness by reducing target expression variance [4]. However, this hypothesis has not yet been tested.

In this study, we investigated the potential roles of miRNAs in buffering inter-individual variation in gene expression in pigs challenged with *Salmonella*. We first tested whether targets of miRNAs show lower expression variance (reduced variation across individual pigs) compared with non-miRNA-targets by controlling for differences in expression levels. Next, we characterized the different patterns of expression variance change between miRNA targets and non-miRNA-targets in response to infection. Further, we identified enrichment of specific functional annotations of miRNA targets with reduced variation of expression after *Salmonella* challenge. Finally, we tested the hypothesis that young miRNAs are likely to have stronger buffering effects compared with old (evolutionarily conserved) miRNAs.

## Results

### Targets of miRNAs on Average showed Lower Expression Variance Compared with Non-miRNA-targets

To investigate the potential buffering roles of miRNAs in response to bacterial infection, we sequenced miRNAs and mRNAs in whole blood from 15 pigs before (day 0) and after *Salmonella* challenge (day 2). The raw count of reads mapped to each miRNA was normalized to counts per million mapped reads (cpm) using edgeR [17] (Table S1). Normalized expression values (FPKM) for each mRNA gene was estimated by Cufflinks 2.0.0 [18] (Table S2). We identified 192 and 15,173 expressed miRNA and mRNA genes, respectively. To provide evidence for the buffering effects of miRNA, we compared the distribution of variation of gene expression between targets of expressed miRNAs and non-miRNA-targets. We used miRanda3.3a [19] and PITA for prediction of miRNA targets in pig (see Methods). miRanda and PITA predicted 7,978 and 10,803 miRNA targets, respectively. We selected the genes predicted by both methods (7,962 targets) for further analysis. For each target gene, we calculated the coefficient of variation (CV) to quantify the expression variation among different samples.

**Figure 1:**
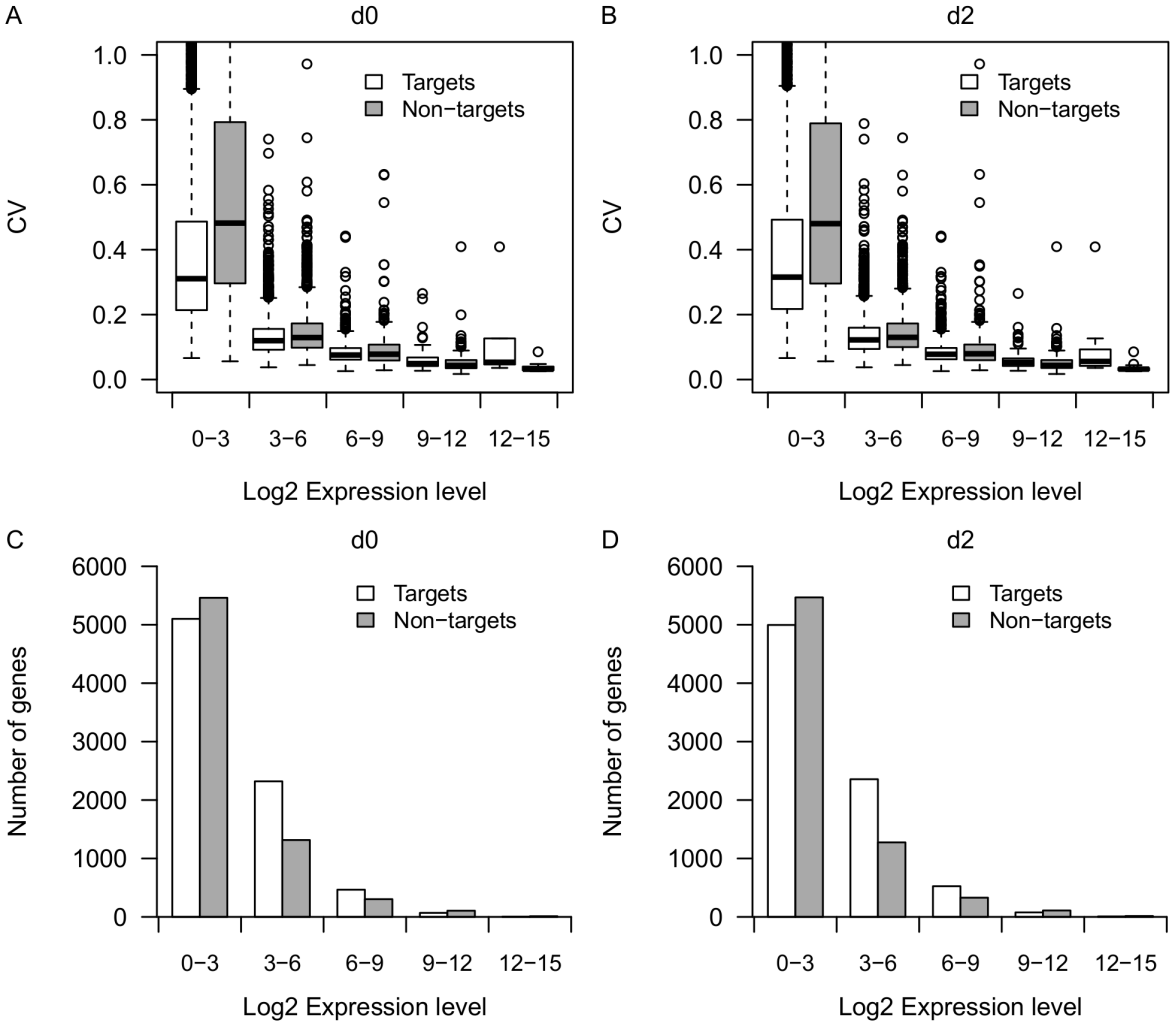
Distribution of CV and number of genes for miRNA targets and non-miRNA-targets. Boxplot of CVs for predicted targets (n = 7962) and non-miRNA-targets (n = 7211) under different expression bins (log2FPKM: 0–3, 3–6, 6–9, 9–12, 12–15) at day 0 (A) and day 2 (B). Barplot of number of genes for predicted targets and non-miRNA-targets under different expression bins (log2FPKM: 0–3, 3–6, 6–9, 9–12, 12–15) at day 0 (C) and day 2 (D).

It has been shown that mRNA expression variance is negatively correlated with mRNA expression level [20], [21]. Thus, in order to control for the difference in expression level, we grouped the genes into 5 bins based on average expression level (log2FPKM: 0–3, 3–6, 6–9, 9–12, 12–15), then we compared the CV between miRNA targets and non-miRNA-targets under each bin (Figure 1A and 1B). Indeed, we observed a negative correlation (r<−0.5, p<2.2e-16) between CV and expression level for both predicted miRNA targets and non-miRNA-targets at day 0 and day 2. Predicted targets on average showed lower expression variation compared with non-miRNA-targets for lowly and medium expressed genes (log2FPKM< = 6) both before and after infection (p<1e-6, Mann-Whitney U test) (Figure 1A and 1B). However, the expression variance between miRNA targets and non-miRNA-targets with extremely high expression levels (log2FPKM>6) was not significantly different (p>0.1, Mann-Whitney U test) (Figure 1A and 1B). Notice that genes with log2FPKM<6 account for more than 90% of the total expressed genes (Figure 1C and 1D). Thus, for the majority of expressed genes, predicted miRNA targets on average showed lower expression variance compared with non-miRNA-targets.

### The Buffering Effect is Stronger for mRNAs Targeted by a Greater Number of miRNAs

**Figure 2:**
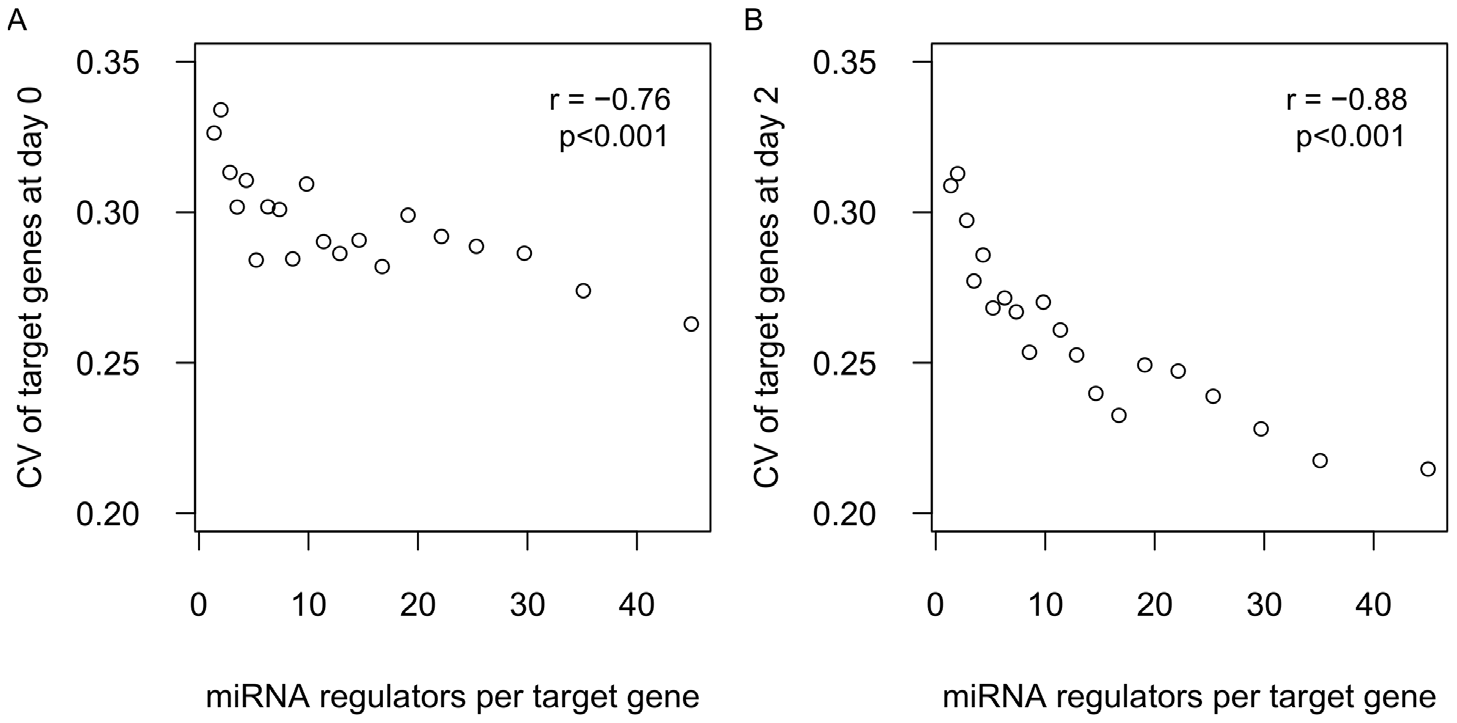
Correlation between CV of target genes and miRNA regulators per gene. All target genes were assigned into 20 equal-sized bins by the number of miRNA regulators. The mean CV and the mean number of miRNA regulators for each bin were plotted using data from day 0 (A) and day 2 (B).

It is known that a single miRNA can regulate hundreds of mRNAs and that a single mRNA can be regulated by multiple miRNAs [22]. In our data, approximately 50% of the genes were targeted by less than 10 miRNAs each and below 1% were targeted by over 50 miRNAs each (Figure S1A). The distribution of target genes per miRNA is provided in Figure S1B. It is possible that the buffering effect is stronger for mRNAs targeted by a greater number of miRNAs. To test this hypothesis, we calculated the correlation between the number of miRNA regulators per target gene and the CV of target expression. We found a strong negative correlation between the CV of target expression and the number of miRNA regulators per target gene at both day 0 (r = −0.76, p<0.001, Figure 2A) and day 2 (r = −0.88, p<0.001, Figure 2B). Further, the decreasing CV with increasing number of miRNA regulators cannot be explained by the previously described tendency of more highly expressed genes to have lower CV, since the genes with more miRNA regulators tend to exhibit lower expression (Figure S2). Taken together, the results suggest that the buffering effect is stronger for mRNAs targeted by a greater number of miRNAs.

### Different Pattern of Expression Variance change between miRNA Targets and Non-miRNA-targets in Response to Infection

**Figure 3:**
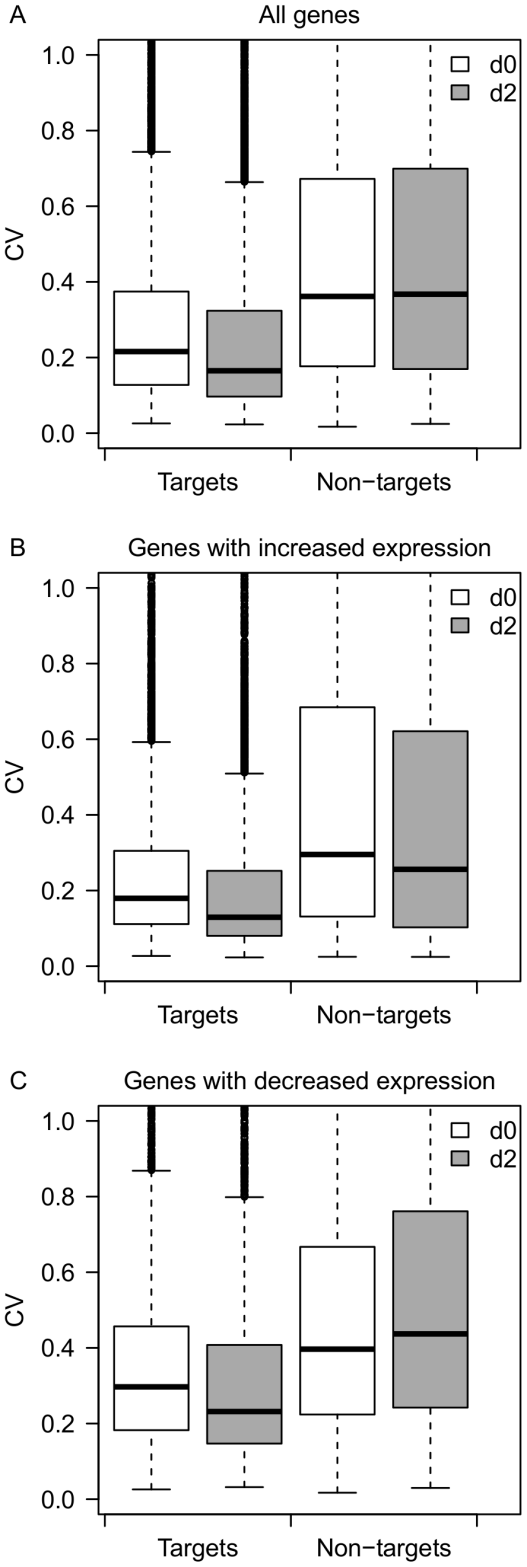
Change of CV in response to infection. (A) Boxplot of CVs for predicted miRNA targets (n = 7962) and non-miRNA-targets (n = 7211) before and after infection. (B) Boxplot of CVs for predicted miRNA targets (n = 2947) and non-miRNA-targets (n = 4029) with decreased expression after infection. (C) Boxplot of CVs for predicted miRNA targets (n = 5015) and non-miRNA-targets (n = 3182) with increased expression after infection.

A previous study found that a number of genes have a significant increase or decrease in expression variance between different biological states (e.g. diseased and non-diseased) [23]. First, we analyzed the expression variance change of miRNA targets and non-miRNA-targets before and after infection. The median CV of predicted miRNA targets showed a 23.6% reduction after infection (p<2.2e-16, Mann-Whitney U test), but non-miRNA-targets showed no significant change (p = 0.5, Mann-Whitney U test) (Figure 3A). Next, we applied the same tests to the genome-wide average expression. The median expression level of both miRNA targets and non-miRNA-targets showed no significant differences after infection (p>0.1). Although the genome-wide average expression did not change significantly, several genes indeed showed increased (log2 fold change<0, n = 6976) or decreased (log2 fold change>0, n = 8197) expression after infection, and this can substantially contribute to the variation of gene expression between different conditions. Thus, to further control for the difference in expression level between day 0 and day 2, we analyzed the CV change separately for genes with increased (log2 fold change<0) or decreased expression (log2 fold change>0) after infection (Figure 3B and 3C, respectively). For non-miRNA-targets, the change of CV was negatively correlated (r = −0.53, p<2.2e-16) with the change of expression levels, which is to be expected given the general relationship between CV and expression. Interestingly, miRNA targets showed on average reduced expression variance regardless of whether their expression is increased or decreased in response to infection. The results indicate that *Salmonella* infection is accompanied by decreased expression variance for miRNA targets but not for non-miRNA targets. Furthermore, this result cannot be explained by the tendency of more highly expressed genes to have lower CV.

### Reduced Expression Variance of Transcripts Encoding RNA Binding Proteins in Response to Infection

We next examined whether any particular gene functions were enriched among the miRNA targets showing substantially reduced (>50%) variation after *Salmonella* infection. As stated earlier, genes with higher expression tend to have lower CV. To avoid including genes with reduced variation solely due to their increased expression, we discarded genes with substantial increased expression (expression fold change>1.2). We identified 759 predicted miRNA targets (expression fold change< = 1.2) that showed more than 50% reduction of CV after infection. We then looked for enriched gene ontology (GO) terms in these genes. In the GO molecular function category, there was an enrichment in RNA binding (FDR<1e-9), and in the biological process category there was an enrichment in RNA processing related terms (FDR<1e-4). Complete GO enrichment results are shown in Table 1.

**Table 1:** GO enrichment of miRNA target genes with reduced variation of gene expression.

GO	Term	Count	FDR
	mRNA processing	34	1.0e-5
Biological Process	mRNA metabolic process	32	1.3e-5
	RNA processing	47	1.1e-5
Molecular Function	RNA binding	66	4.1e-10
	Acid-amino acid ligase activity	24	1.4e-4

**Figure 4:**
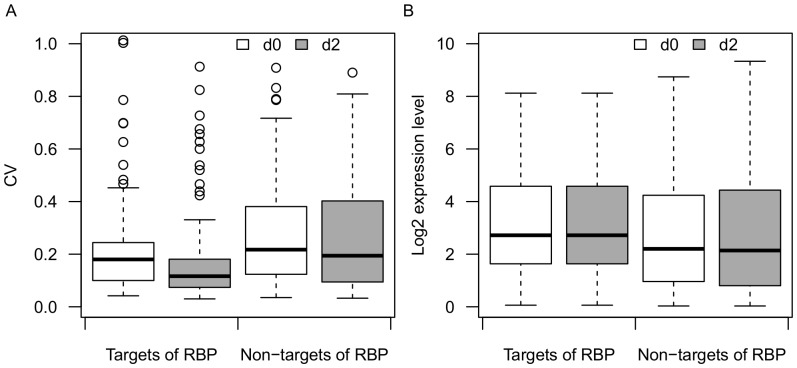
Distribution of CV and expression levels for RBPs. (A) Boxplot of CVs for predicted miRNA targets of RBPs (n = 179) and non-miRNA-targets of RBPs (n = 122) before and after infection. (B) Boxplot of mean expression level for predicted miRNA targets of RBPs (n = 179) and non-miRNA-targets of RBPs (n = 122) before and after infection.

Next, we compared the distribution of CV and expression level between miRNA targets and non-miRNA-targets of RNA binding proteins (RBPs). miRNA targets of RBPs demonstrated significantly reduced variance of expression after *Salmonella* challenge (p = 3.65e-07, Mann-Whitney U test) (Figure 4A). However, non-miRNA-targets of RBPs also showed lower but not significant (p = 0.22, Mann-Whitney U test) change of expression variance (Figure 4A). The difference in expression level of both miRNA targets and non-miRNA-targets of RBPs between day0 and day2 was not significant (p>0.1, Mann-Whitney U test) (Figure 4B). Furthermore, miRNA-targeted RBPs showed lower expression variance than non-miRNA-targets of RBPs at d0 and d2 (p = 0.004 and 6.59e-7, respectively, Mann-Whitney U test) (Figure 4A). Taken together, the results suggest that RBPs in general show reduced expression variance following infection, but that the level of reduction is more dramatic for RBPs targeted by miRNAs.

### Targets of Young miRNAs showed Lower Expression Variance Compared with Targets of Old miRNAs

**Figure 5:**
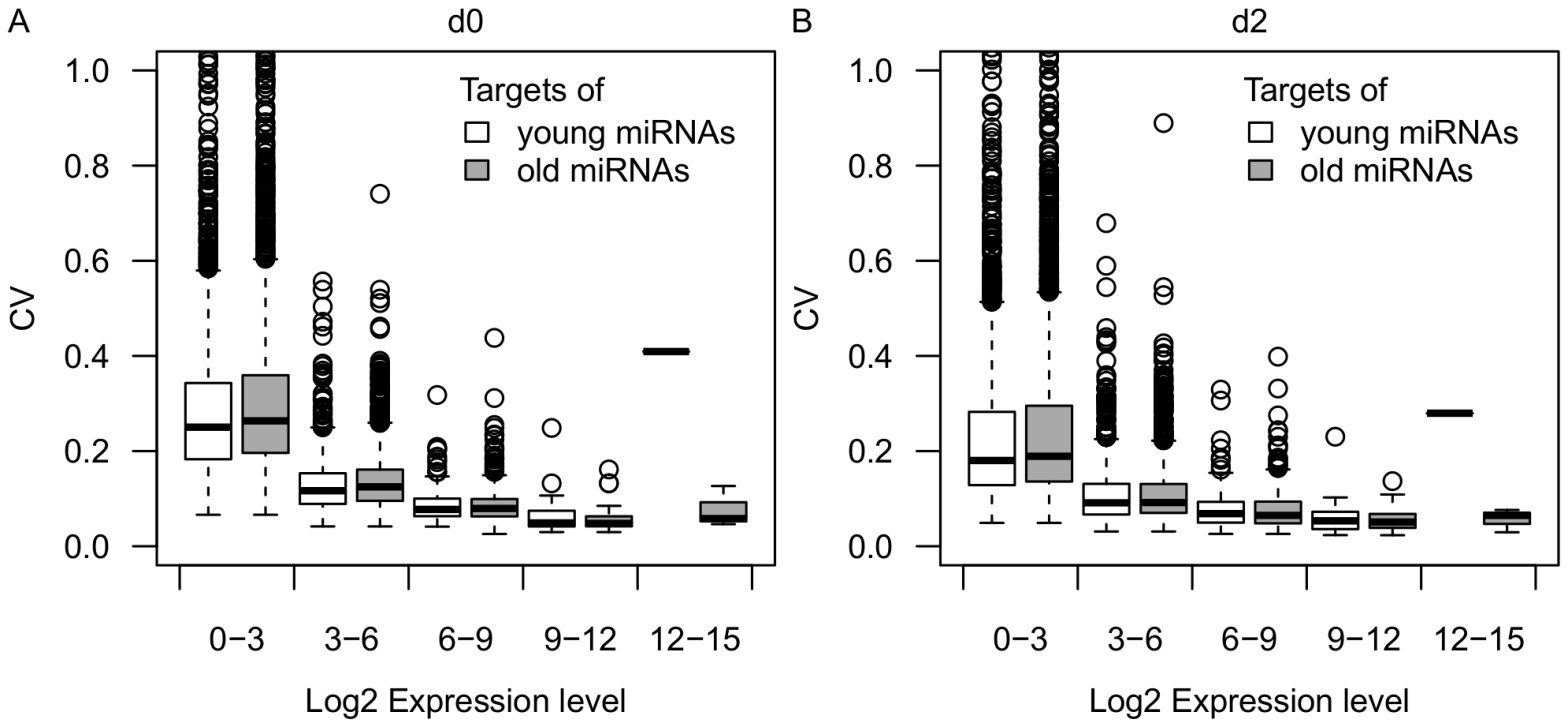
Distribution of CV for targets of young and old miRNAs. Boxplot of CVs for targets of young miRNAs (n = 4162) and targets of old miRNAs (n = 7415) under different expression bins (log2FPKM: 0–3, 3–6, 6–9, 9–12, 12–15) at day 0 (A) and day 2 (B).

It has been hypothesized that the dual functions of miRNAs may represent two stages in the evolution of miRNAs, with young (less-conserved) miRNAs playing a greater role in expression buffering than old miRNAs (evolutionarily conserved) [4]. We tested this hypothesis by analyzing the difference of CV between targets of young and old miRNAs. To identify relatively young and old miRNAs, six animal genomes (human, dog, cattle, mouse, rat and chicken) were used for conservation analysis. We considered pig-specific miRNAs to be relatively young miRNAs (n = 9) and miRNAs conserved in all six species as old miRNAs (n = 62). Indeed, targets of young miRNAs on average showed significantly lower expression variance compared with targets of old miRNAs for lowly and medium expressed genes (log2FPKM< = 6) at both 0 and 2 dpi (p<0.05, Mann-Whitney U test) (Figure 5A and 5B). Thus these results support the hypothesis that young miRNAs tend to have stronger buffering effects compared with old miRNAs.

## Discussion

One of the most remarkable features of biological systems is their inherent robustness against external perturbations [24]. miRNAs have been hypothesized as canalizing genes [4], [5]. However, when there are no input perturbations, the buffering function might not be easily noticeable. Such states or noises requiring this function could be present at various developmental stages or under stress conditions including severe infection, chemical or radiation treatment. Here, we showed that miRNA targets had lower expression variation compared with non-miRNA-targets for lowly and medium expressed genes both before and after infection. It has been shown that lowly expressed genes have higher expression variation [21]. Thus, our results point to the importance of a buffering effect of miRNAs for lowly and moderately expressed genes. Further, we found that the CV of target genes is negatively correlated with the number of miRNA regulators. Thus, multiple miRNAs may have additive buffering effects on a single target gene.

From an evolutionary perspective, it has been hypothesized that young miRNAs may play a greater role in expression buffering than old miRNAs [4]. The results of our analysis on the difference in CV between targets of young miRNAs and targets of old miRNAs support this hypothesis. As adjusting the mean expression levels of several target genes is generally deleterious, young miRNAs are not likely to increase fitness by dramatically modulating the expression levels of a large number of target genes when they are born. Instead, they may confer a selective advantage by buffering the variances in gene expression. After the new miRNAs become fixed into the genome, the tuning function (regulating the mean expression level of targets) may evolve gradually through random drift [25] or positive selection.

It has been shown that the actions of miRNAs and TFs are often highly coordinated and connected as network motifs [26]. One of the most important motifs is the miRNA-mediated IFFL in which the genes that encode an miRNA and that miRNA’s target mRNA are both positively regulated by a TF, and simultaneously the target gene is negatively regulated by the miRNA. Further, it has been shown that IFFLs can reduce the stochastic noise in expression of such target genes [9]. According to simulation studies, the degree of buffering within an IFFL depends on the expression levels of TFs and miRNAs and on the suppression strengths of the miRNAs [9]. Further, a previous study [27] analyzed the relationship between TFs and miRNAs in gene regulation networks and found that miRNAs predominantly target positive regulatory motifs. It has been reported that the genes with more TF binding sites have a higher probability of being targeted by miRNAs and have more miRNA-binding sites on average [28]. Our results in light of previous studies suggest that the regulatory networks consisting of miRNAs and TFs may contribute to reduce the expression variance of miRNA targets in pigs.

Another interesting finding of this study is that *Salmonella* infection is accompanied by decreased expression variance for miRNA targets but not for non-miRNA targets. Even for down-regulated genes after infection, miRNA targets still showed reduced variation, suggesting a major role of miRNAs in conferring robustness to infection. From a network perspective, one explanation of reduced variation of miRNA targets is that the transcriptional network may be re-wired more tightly after infection. For example, expression of additional miRNAs and TFs following *Salmonella* infection may re-wire the existing networks or establish new network modules specific to the infection condition. Further, the expression of miRNAs and TFs may change to their optimal level to buffer variations more efficiently during *Salmonella* infection. An extensive survey of the expression of TF, miRNA and experimentally verified targets within known regulatory networks under different conditions in model species might be informative about how miRNA affect the noise of target gene expression.

RBPs are the key regulators of noise buffering in numerous downstream cellular processes [29]. Due to the central role of RBPs, alteration in their expression has been reported to be the cause of several human diseases [30]. Previous studies showed that RBPs have very little variation and tend to be highly connected in the TF regulatory network [31]. Several RBPs were found to be regulated by miRNAs in tumor models [32]. However, the potential role of miRNAs in buffering expression variation of RBPs has not been investigated previously. Here, we show evidence that miRNAs may also contribute to buffering expression variance of RBPs. Transcripts encoding RBPs in general showed reduced expression variance following the infection, but the level of reduction is more dramatic for RBP RNAs targeted by miRNAs. Further, RBPs were also found to play an important role in immune response [33], [34]. For example, HuR (an RBP gene) can maintain inflammatory homeostasis by controlling macrophage plasticity and migration in mice [33]. In our study, we found that the CV of *HuR* in pig decreased from 0.12 to 0.06 after infection. Six miRNAs (miR-144, miR-145, miR-2483, miR-27b, miR-296 and miR-324) may target *HuR* based on our predictions. A more comprehensive analysis of known miRNA and RBP interactions in human or mouse cell models might be helpful to better understand miRNA-mediated regulation of RBP homeostasis.

The current study and previous ones [13]–[15] have focused on the buffering effects of miRNAs on inter-individual variation in gene expression. However, little is known about the buffering effects of miRNAs on expression variation among single cells. The availability of data [35], [36] generated using the recently developed single cell RNA-seq technology would make it possible to investigate miRNA buffering effects on cell-to-cell variation in gene expression.

In conclusion, these findings point to the importance of buffering effects of miRNAs, and suggest that the reduced expression variation of RBPs may play an important role in response to *Salmonella* infection. A more comprehensive understanding of the miRNA-mediated regulation of expression variation could improve our ability to predict miRNA targets and biological functions, and provide insight into the mechanistic basis for their buffering activities.

## Materials and Methods

### miRNA and mRNA Sequencing of Whole Blood Samples from Pigs

Fifteen pig blood samples were used for miRNA and mRNA sequencing before and after *Salmonella* challenge (2 dpi). Peripheral whole blood (approximately 2.5 mL) was collected from the jugular vein of pigs, into PAXgene Blood RNA tubes (BD, Cat. No. 762165) and processed according to the manufacturer’s instructions. Total RNA (1.5 μg for each sample) was used to construct miRNA and mRNA libraries using the TruSeq Small RNA and mRNA Sample Preparation Kit(Illumina, San Diego, CA), respectively, according to the manufacturer’s instructions. Globin reduction treatment with pig globin specific oligonucleotides was performed using a modified Affymetrix globin reduction protocol [37]. Sequencing was performed on the HiScan SQ system (Illumina) using the TruSeq™ SBS Kit v3 (50 cycels, Illumina). All procedures involving animals were approved by the USDA-ARS-NADC Animal Care and Use Committee (approval ID: ACUP #3586).

### Quantification of miRNA and mRNA Expression in Pig Samples

miRNA reads were mapped onto known pig miRNAs (miRBase 19.0) using miRDeep2 [38]. mRNA reads were mapped onto pig genome using Tophat1.4.0 [39]. The raw count of reads mapped to each miRNA was normalized to counts per million mapped reads (cpm) using edgeR [17]. Normalized expression value (FPKM) for each mRNA gene was estimated by Cufflinks 2.0.0 [18]. After that, normalized expression levels were log2 transformed.

### Calculation of Variation of Gene Expression

We analyzed the difference of expression variation between miRNA targets and non-targets as described by Lu [15]. We used the coefficient of variation (CV), which is computed for each gene by dividing the standard deviation of its expression measures across a sample population by its average expression, to quantify the extent of expression variation.

### miRNA Target Prediction

To identify high-confidence miRNA targets, we used both miRanda3.3a [19] and PITA. Alignment score> = 145 and energy< = −10 kcal/mol were used for miRanda as suggested by Zhang [40]. PITA determines the change in free energy (ddG) necessary for the binding to occur. ddG score< = −10 was used for PITA [41]. Only miRNA targets predicted by both methods were used.

### Gene Ontology Analysis and Identification of RBPs

The genes with more than 50% reduced expression variation after infection were subjected to GO enrichment analysis using the web-based functional annotation tool, DAVID version 6.7 (http://david.abcc.ncifcrf.gov/) [42]. We downloaded human RBPs from the RNA binding protein database (RBPDB) (http://rbpdb.ccbr.utoronto.ca/) [43]. We used pig orthologous as RBPs identified in pigs.

## Supporting Information

Figure S1 **Distribution of miRNA regulators and target genes.** (A) The distribution of the number of miRNA regulators per gene. (B) The distribution of the number of target genes per miRNA.
(TIF)

Figure S2 **Correlation between expression of target genes and miRNA regulators per gene.** All target genes were assigned into 20 equal-sized bins by the number of miRNA regulators. The mean expression level and the mean number of miRNA regulators for each bin were plotted using data from day 0 (A) and day 2 (B).
(TIF)

Table S1 **Normalized miRNA expression level in pigs.**
(XLSX)

Table S2 **Normalized mRNA expression level in pigs.**
(XLSX)
